# P53 expression in various types of hydropic placentas (through ploidy analysis as a complementary tool in diagnosis of samples)

**DOI:** 10.22088/cjim.10.2.205

**Published:** 2019

**Authors:** Alireza Khooei, Fatemeh Atabaki Pasdar, Alireza Fazel, Mahmoud Mahmoudi, Mohammad Reza Nikravesh, Shahrzad Daneshmand Shahbazian

**Affiliations:** 1 Department of Pathology, Emam Reza Hospital, Mashhad University of Medical Sciences, Mashhad, Iran; 2 Department of Anatomical Sciences, Faculty of Medicine, Urmia University of Medical Sciences, Urmia; 3 Immunology Research Center, Bu Ali Research Institute, Mashhad University of Medical Sciences, Mashhad, Iran; 4 Department of Anatomy and Cell Biology, faculty of Medicine, Mashhad University of Medical Sciences, Mashhad, Iran; 5 Department of Biomedical Engineering, School of Electrical Engineering, Iran University of Science & Technology (IUST), Tehran, Iran

**Keywords:** Hydatidiform Mole, Abortion, Immunohistochemistry, P53 Antigen

## Abstract

**Background:**

Placentas characterized by hydropic swelling of chorionic villi occur in a spectrum of pathological conditions including hydropic abortion (HA), partial hydatidiform mole (PHM) and complete hydatidiform mole (CHM). The purpose of this study was to investigate whether the expression of p53 tumour suppressor protein could differentiate these various types of hydropic placentas.

**Methods:**

p53 immunohistochemical staining was performed in 19 molar (8 PHM and 11 CHM) and 10 non-molar (HA) formalin-fixed, paraffin-embedded tissue samples. Ploidy analysis using flow cytometry was performed as a complementary tool in diagnosis of samples.

**Results:**

DNA histograms obtained from all samples had confirmed diploidy in HAs and CHMs and triploidy in PHMs. p53 immunoreactivity was assessed in villous cytotrophoblasts, syncytiotrophoblasts and stromal cells. The p53 positive reaction was predominantly observed in the nuclei of cytotrophoblastic cells and rarely in stromal cells, no reaction was seen in syncytiotrophoblasts. The mean percentage of p53 positive cells were 6.10±3.75 for HA, 25.87±13.4 for PHM and 39.83±18.76 for CHM. There was a significant difference in P53 immunoreactivity of cytotrophoblastic cells between CHM and HA (*P*<0.001), and between PHM and HA (*P*=0.004). There was no significant difference in immunohistochemical reactivity between CHM and PHM (*P*=0.068).

**Conclusion:**

This study confirms that p53 immunostaining may be helpful in distinguishing complete and partial hydatidiform mole from hydropic abortion, but not complete hydatidiform mole from partial hydatidiform mole.

Gestational trophoblastic disease (GTD) comprises a group of disorders that arise from placental trophoblastic tissue after abnormal fertilization and may follow a hydatidiform mole (HM) or a nonmolar pregnancy. These disorders include premalignant and malignant conditions. HMs represent premalignant condition. The malignant form of the disease is known as gestational trophoblastic neoplasia (GTN). GTN is comprised of four histologic subtypes: invasive mole, choriocarcinoma, epithelioid trophoblastic tumor and placental-site trophoblastic tumor (1, 2). HM or molar pregnancy is the most common form of GTD; this includes both partial hydatidiform mole (PHM) and complete hydatidiform mole (CHM). The importance of such a condition derives from its potential for persistent trophoblastic disease or GTN (3). The incidence of molar pregnancy varies geographically, being highest in Asian countries (4).

HM is an abnormal pregnancy characterized by hydropic swelling of placental villi and trophoblastic hyperplasia (5), Placentas characterized by hydropic swelling of chorionic villi occur in a spectrum of pathologic conditions including hydropic abortion (HA), partial hydatidiform mole, and complete hydatidiform mole. Degenerative changes in a nonmolar placenta (so-called "hydropic abortion") is a phenomenon where numerous cystic spaces are formed within the placenta which is often accompanied by placental enlargement. It can occur in a first trimester pregnancy loss. In this situation the serum beta HCG tended to be low and would show a decline. Sonographic appearances can sometimes mimic gestational trophoblastic disease (6).

Accurate diagnostic classification of hydropic placentas is important as the risk of persistent gestational trophoblastic diseases or GTN is different among the three entities (7). Whereas hydropic abortion is completely benign, hydatidiform moles have a significant risk for developing persistent gestational trophoblastic disease, with a higher incidence in patients with complete hydatidiform mole (10-30%) than in patients with partial hydatidiform mole (0.5-5%) (8). Histologic examination is the main tool in the diagnosis of molar pregnancies. However, there is considerable overlap in the histologic features between molar and non-molar pregnancies and between complete hydatidiform mole (CHM) and partial hydatidiform mole (PHM), resulting in significant inter-observer and intra-observer variability in the diagnosis (9, 10). Recently, pathologists have relied on molecular techniques, such as DNA flow cytometry, chromosome in situ hybridization, and polymerase chain reaction-based genotyping or HLA typing, which by showing DNA content differences, help to correctly identify the hydropic placentas (11). However, the molecular methods are technically difficult, relatively expensive and time consuming. Notably, the immunoistochemistry (IHC) plays a very important role in the differential diagnosis between molar disease and non-molar abortions (12). It was evident that gestational trophoblastic disease exhibits an increased apoptotic activity when compared with non-molar placentas (13), moreover, apoptosis appears to be related closely to the risk of GTN after CHM. It has been found that a low apoptotic index is associated with a higher risk of GTN (14). 

P53 is known as a tumor suppressor gene which encodes a nuclear phosphoprotein and its mutation seems to be involved in many human cancer pathogenesis (15). The tumor-suppressor protein p53 is an important inducer of apoptosis (16). Several studies have revealed that overexpression of p53 is involved in the pathogenesis of GTD (17, 18). The present study was carried out to evaluate the expression pattern of p53 in HAs, PHMs and CHMs, and to assess the value of this marker in differential diagnosis of the three entities.

## Methods

**Case Selection: **Formalin-fixed, paraffin-embedded gestational products from 29 patients, including 11 complete hydatidiform moles, 8 partial hydatidiform moles and 10 hydropic spontaneous abortions diagnosed in the Emam Reza and Qhaem Departments of pathology, affiliated to Mashhad University of Medical Sciences, were gathered. The present study has been approved by the Ethics Committee of Mashhad University of Medical Sciences.

Tissue sections of the specimens were stained with routine hematoxylin-eosin and histopathologically reviewed by the pathologist using published criteria (19), Diagnosis of three entities was confirmed by ploidy analysis using flow cytometry in all samples and confirmed diploidy in spontaneous abortions and complete moles, and triploidy in partial moles (20).

**Flow Cytometry: **Flow cytometric DNA analysis was performed on formalin-fixed, paraffin-embedded tissue blocks. The selection criterion for the blocks was the presence of both placental and maternal (decidual) tissue in approximately such amounts that representative DNA histograms could be anticipated. Maternal tissue had to be present as the internal diploid control. One 50 μm section of each block was placed in 10 ml glass centrifuge tubes and dewaxed using two changes of xylene, 3 ml for 10 min at room temperature, and then rehydrated in a sequence of 3 ml of 100%, 95%, 75%, and 50% ethanol for 10 min each at room temperature with centrifugation and decantation of the supernatant after each step. The tissues were then washed twice in distilled water and re-suspended in pepsin solution (1 mL of 0.05% pepsin in 0.9% NaCl, pH 1.5) at 37°C for 45-60 minutes with intermittent mixing using a vortex. The reaction was stopped with cold PBS and the samples were washed twice with phosphate buffered saline (PBS).The resulting cell suspension was washed twice with PBS. After addition of RNase to remove any nuclear or residual cytoplasmic RNA, and propidium iodide, ploidy was determined by flow cytometry using facscalibur flow cytometer (Becton-Dickinson). The data were analyzed with use of the computer program Lysys II Software (Becton-Dickinson, Mountain View, CA, USA) (20).

**Immunohistochemistry: **5μm thick sections were cut and incubated for 60 min at 60ºC, then the sections were deparaffinized in xylene and rehydrated in a descending ethanol series. Endogenous peroxidase activity was blocked by a 20 minute treatment with three percent hydrogen peroxidase in phosphate-buffered saline (PBS). The slides were then washed twice in PBS, pH 7.4 and subsequently transferred to retrieval buffer (10-Mm sodium citrate buffer, pH 6.0) and heated in a microwave oven (at a power of 700 W). The slides were left to cool at room temperature, then were incubated with mouse monoclonal antibody for 30 min at room temperature (p53: prediluted (ready to use), Clone DO-7, N1581, Dako, Glostrup, Denmark). Later the sections were rinsed in PBS and incubated with polymer-based Envision (Dako Cytomation, Glostrup, Denmark). The chromogenic reaction was performed by 3, 3-diaminobenzidine (DAB), (Dako Cytomation, Glostrup, Denmark). The sections were then counterstained with Mayer̛s hematoxylin. The sections of colon cancer were used as a positive control for p53, and negative controls were stained by skipping primary antibody incubation (21, 22). Evaluation of protein expression was carried out. All immunostained sections were independently examined by the same two observers with a ×400 objective under the light microscope (Olympus BX-51, Olympus, Tokyo, Japan), while they did not know about the slide diagnosis, therefore the analysis was double-blind. A staining is considered positive, when the cells show a positive nuclear staining, immunoexpression analyses for villous cytotrophoblasts, syncytiotrophoblasts and stromal cells, commenced from the field with most staining, separately by counting 100 cells of each population per slide, (23, 24).

**Figure 1:**
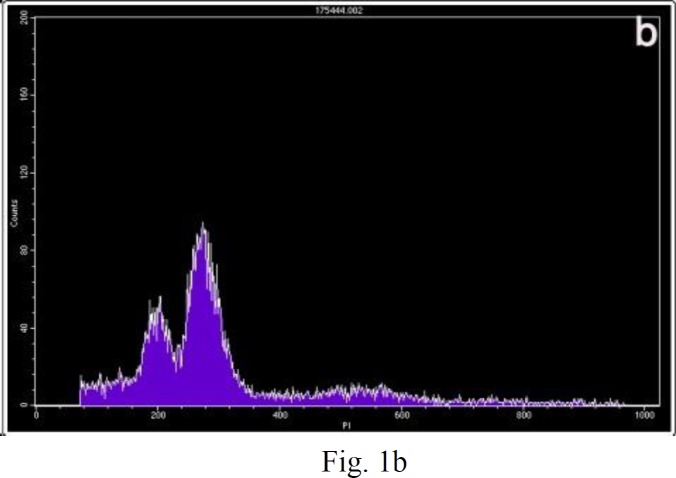
Examples of the two kinds of DNA histograms. Vertical axis, number of counted events; horizontal axis, channel number, representing the relative DNA content. (a) Normal diploid DNA histogram. One high peak is considered to be diploid maternal and placental cell populations. (b) DNA histogram expressing triploidy

The first peak represents maternal diploid cells and the second peak represents placental cells with a triploid DNA content (20). Statistical analyses were conducted using Kolmogorov-Smirnov, ANOVA and Tukey’s HSD tests. The results were expressed as mean±SD. The differences were considered statistically significant at a p-value less than 0.05. All statistical tests were performed using SPSS software.

## Results

**Figure 2:**
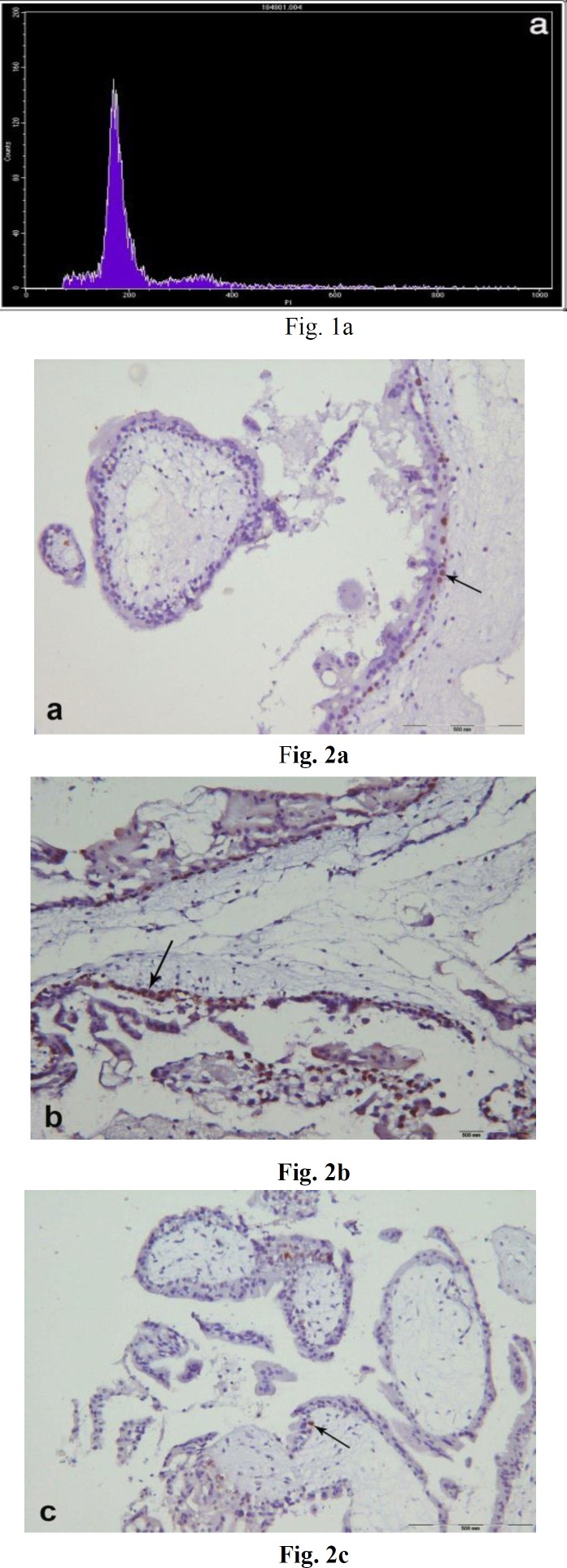
Immunoreactivity with p53 in partial hydatidiform mole (a), complete hydatidiform mole (b) and hydropic abortion (c), which confined to the nuclei of cytotrophoblasts. (arrow), (counterstained with Mayer̛s hematoxylin original magnification x 400)

**Figure 3:**
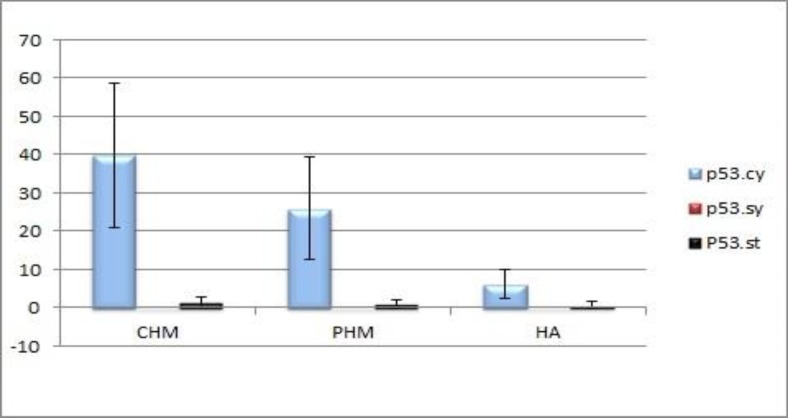
The percentage of p53 positive cells in various groups (Mean±SD)

DNA histograms were obtained from all samples. Examples of the DNA histograms expressing diploidy in spontaneous abortions and complete moles and triploidy in partial moles are shown in (fig.1a-b). p53 immunoreactivity was assessed in villous cytotrophoblasts, syncytiotrophoblasts and stromal cells. Positive cells were found to be restricted mostly to the villous cytotrophoblasts, while syncytiotrophoblasts showed an absence of immunostaining for p53, and occasional weak nuclear staining was seen in the stromal cells (fig. 2a-c). The percentage of p53 positive cells are shown in (fig. 3). There was a significant difference in p53 immunoreactivity with cytotrophoblastic cells between complete hydatidiform moles and hydropic abortions and also between partial hydatidiform moles and hydropic abortions,  There was no significant difference in immunohistochemical reactivity between CHM and PHM (P=0.068). The results of statistical analyses are summarized in (table 1).

**Table 1:** Results of statistical analysis to compare p53 expression between groups

**Groups**	PHM, HA	CHM, HA	CHM, PHM
P.Value	P=0.004	P<0.001	P=0.068

CHM: Complete Hydatidiform Mole;

PHM: Partial Hydatidiform Mole;

HA: Hydropic Abortion

## Discussion

About 50% of GTN follows molar pregnancy (25), whereas hydropic abortion is completely benign (8). The histologic separation of hydropic abortions from partial moles and of partial moles from complete moles may be difficult. Although diagnostic criteria are established, there is considerable intra and inter-observer variability when using gross and microscopic findings alone (9, 10). Studies have recently shown that immunohistochemistry for various markers is useful for confirming the diagnosis and is a complementary method to pathologic interpretation (26). The value of immunohistochemical analysis of paternally imprinted, maternally expressed p57 gene for improving the diagnosis of hydatidiform moles has been demonstrated in a number of recent studies (27, 28). However, p57 immunohistochemistry can identify complete hydatidiform moles (androgenetic diploidy) by the lack of p57 expression but cannot distinguish partial hydatidiform moles (diandric monogynic triploidy) from non-molar (biparental diploidy) specimens. The tumour suppressor p53 plays a central role in protection against DNA damage, uncontrolled proliferation and neoplastic transformation, primarily by inducing cell cycle arrest or apoptosis (29). 

In this study, p53 immunohistochemical staining was performed in 19 molar (8 PHM and 11 CHM) and 10 non-molar (HA) formalin-fixed, paraffin-embedded tissue samples to assess the value of this marker in differential diagnosis of these three entities. The p53 positive reaction was predominantly observed in the nuclei of cytotrophoblastic cells and rarely in stromal cells, while syncytiotrophoblasts showed an absence of immunolocalization. This is consistent with previous studies performed by Qiao et al. (30), Halperin et al. (31) and kale et al. (16). Cytotrophoblast is the trophoblastic stem cell, whereas syncytiotrophoblast is the terminally differentiated cell (32), therefore p53 may be an indicator of proliferative activity. Based on the data, obtained in the present study, there was a significant difference in P53 immunoreactivity of cytotrophoblastic cells between CHM and HA and between PHM and HA, although there was a difference between CHM and PHM, but this did not reach statistical significance. This is consistent with previous studies performed by Kheradmand et al. (33) and Al-Bozom et al. (34). It has been reported that p53 gene mutation is rare in complete hydatidiform mole and trophoblastic tumours (31, 35). Cheung et al. reported a positive correlation between p53 and Ki-67 proliferation index in trophoblastic tissues of hydatidiform moles (36). Hence, p53 overexpression may be a reflection of the higher proliferation capacity of the trophoblastic cells in molar tissues. A possible role of expression of the p53 protein in proliferative trophoblastic tissues is an attempt to modulate the excessive proliferative activity in trophoblastic cells (37). On the other hand, Halperin et al. evaluated the expression of the p53 and apoptosis in GTD and normal placenta and showed that the percentage of apoptotic cells demonstrated a significant increase in HMs compared with normal placenta and also significant overexpression of p53 in HMs compared with normal placenta, they concluded that p53 overexpression in hydatidiform moles could be the result of upregulation of apoptosis (31). A recent study reported significantly higher p53 expression in CHMs compared with the PHMs and HAs (26), whereas based on our findings, although there was a difference between CHMs and PHMs, this did not reach the statistical significance. This discrepancy may be due to the use of different antibody clones or retrieval methods, furthermore, in our study ploidy analysis using flow cytometry was performed for the confirmation of the histologic diagnosis of samples. 

In conclusion, this study confirms that p53 immunostaining may be helpful in distinguishing complete and partial hydatidiform mole from hydropic abortion, but not complete hydatidiform mole from partial hydatidiform mole.
